# Notes regarding the next era for the *Journal of Clinical Microbiology*

**DOI:** 10.1128/jcm.01647-25

**Published:** 2025-12-08

**Authors:** Romney M. Humphries

**Affiliations:** 1 Vanderbilt University Medical Center 12328, Nashville, Tennessee, USA

**Keywords:** equity, editor in chief, editorial

## EDITORIAL

“All will benefit from the dissemination of the new knowledge and the valuable experiences reported by the devoted scientists, who proudly call themselves Clinical Microbiologists.”

This quote is from the front matter of the inaugural issue of the *Journal of Clinical Microbiology* (JCM), published on 1 January 1975. While short, the congratulatory message from Analytab Products Inc. to the American Society for Microbiology and future readers of the *Journal of Clinical Microbiology* inspires. Clinical microbiologists, physicians, patients, and the public have all benefited from the knowledge gleaned from publications in JCM over the first 50 years of its history. Examples of seminal works housed in our archives include the first description of pulsed-field gel electrophoresis for bacterial strain typing (1), which ushered in an era of molecular strain typing and revolutionized epidemiology and infection control. Boom and colleagues published their method for nucleic acid extraction using guanidinium thiocyanate and silica particles (2) in JCM in 1990. The Nugent scoring system for the diagnosis of bacterial vaginosis (3) and the first applications of PCR methods for the detection of influenza A (4), enterovirus (5), West Nile virus (6), dengue (7), *Mycobacterium tuberculosis* (8), and *Staphylococcus aureus* (9), among many other pathogens, were also published in the *Journal of Clinical Microbiology*. Clinical microbiology laboratories across the world use methods developed from the building blocks of these works, benefiting the care of countless patients. The journal has also highlighted important epidemiological findings, such as the emergence and spread of methicillin-resistant *S. aureus* with novel staphylococcal cassette chromosome *mec* type IV in the community (10), as well as the first description of the granulocytotropic rickettsial pathogen *Anaplasma phagocytophilum* (11) and vancomycin-intermediate *S. aureus* in U.S. hospitals (12). The works published in the *Journal of Clinical Microbiology* shaped our collective response to the COVID-19 pandemic, with an impressive 289 articles published over 3 years and over 19,500 citations (Dimensions database, https://app.dimensions.ai, accessed 27 August 2025). Prior to the COVID-19 pandemic, methods to detect other viruses of pandemic potential, including novel swine origin influenza A (H1N1) (13, 14), Middle East respiratory syndrome (15), and severe acute respiratory syndrome coronavirus (16) were described in the journal.

The evolution of our field of clinical microbiology is well documented in our archives. At times, adapting to change has led to debate among colleagues, as new techniques and data challenged historical dogmas for the practice of laboratory medicine. The journal has become an important platform for this debate. Recent examples include our community’s grappling over how to best approach the inexorable tide of taxonomic updates for microorganisms of medical importance (17) or replacing traditional cultures with culture-independent tests (18–21).

Against this backdrop, it is my great honor to take on the role of Editor in Chief for the *Journal of Clinical Microbiology*. It is also a humbling and daunting prospect to lead the academic home for clinical microbiology. Dr. McAdam’s advice to me for the role he held for the last 10 years was to “be bold and make major change.” This is a rather intimidating directive, lessened only by the knowledge that the journal has grown and flourished under his direction and is in excellent shape. The *Journal of Clinical Microbiology* has become the premier journal for clinical microbiology and is among the top that publish microbiology content. The Editors and Editorial Board members recruited by Dr. McAdam are first-rate scientists and clinical microbiologists, and I have been continually impressed by their acumen and instinct; the excellent science published in the journal is fully attributed to their dedication and hard work. I am also supported by an amazing staff at ASM Journals—their patience with me as I fumbled through the first months in this role has been impressive. Through the collective wisdom of this group, the future of the *Journal of Clinical Microbiology* is starting to take shape.

First, the core mission of JCM will not change. Publication of the highest quality studies in clinical microbiology and educational content in the brief case, photo quiz, and minireview features are foundational to our efforts over the next 5–10 years. A key area of expansion is our global inclusivity, and we will be exploring strategies to publish diverse perspectives and data from countries and regions that are historically poorly represented in the journal. This will be accomplished through the addition of Editors and Editorial Board members from outside the United States, including the newly appointed Mark Nicol (The University of Western Australia). In addition and as promised (22), I am delighted that Dr. Elitza Theel has agreed to take on the role of Editor for Diagnostic Access and Equity and will oversee a new journal section under this same title. Articles published by the *Journal of Clinical Microbiology* are read by policymakers and standards developers, and it is imperative that the journal develop the means to ensure our content is equitable, as infectious disease is a global challenge that affects all humans. Diagnostic innovations outside North America and Europe, married with epidemiological and pragmatic studies from low- and middle-income countries, will enrich the journal. Dr. Theel’s research has focused on this work, and I am excited to see how she shapes this strategic item.

A criticism I have encountered for JCM, particularly during my time in industry, is a perceived lack of acceptance for industry-led publications in the journal. Development of journal features, such as the recent “innovative diagnostic methods,” has been a good step towards remedying this concern, although further clarification for this feature is needed. To this end, the scope of the journal has been revised slightly to better highlight the content we are interested in. Specifically, studies of interest include those that are early assessments of novel methods, but these must have sufficient data to demonstrate reasonable proof of concept. In addition, studies that evaluate the regulatory science, including standardization of reference methods, development of composite references, and novel mechanisms to perform clinical trials are welcome.

The widespread use of preprints was enhanced by the COVID-19 pandemic and has left some questioning the continued role of peer review and academic publishing (23). These individuals suggest journals become focused on content discovery by readers as much as content publication, a concept we will be exploring with the *Journal of Clinical Microbiology*. After all, the primary role of the academic journal is scientific communication—increased frequency for the Editors in Conversation podcast, editorials, and commentaries are planned. In addition, expansion of collated article collections will occur.

Finally, expansion of the journal’s content while maintaining the highest standards is a key strategic imperative. New additions to the scope of JCM, all effective as of this publication, include:

– Studies that evaluate equitable approaches to diagnostic testing across diverse populations and geographies, including pathogens that disproportionately affect underserved populations and diagnostic innovations designed to serve low-resource or remote settings.
– Studies that evaluate the characteristics and clinical impact of specific microorganisms as they relate to laboratory testing, including emerging pathogens, antimicrobial susceptibility profiles, and the pathogen’s impact on specific populations.
– Expansion of epidemiology studies to beyond just those that describe novel laboratory methods for epidemiological evaluations.

This past year has undoubtedly been challenging for science and academic medicine. Access to medical misinformation is high, and anti-science sentiments are pervasive. Poorly conducted or fraudulent studies are being used to justify political means (24). The work we do is challenging, often underfunded, and almost always eked out on evenings and weekends or rare breaks in busy clinical schedules. Nonetheless, I call on clinical microbiologists to persevere. Observation, experimentation, and importantly, publication of your findings lead to real change for patients and public health, globally, and are the best weapon we have in these challenging times.

## References

[1] Tenover FC, Arbeit RD, Goering RV, Mickelsen PA, Murray BE, Persing DH, Swaminathan B. 1995. Interpreting chromosomal DNA restriction patterns produced by pulsed-field gel electrophoresis: criteria for bacterial strain typing. J Clin Microbiol 33:2233–2239. doi:10.1128/jcm.33.9.2233-2239.1995 7494007 PMC228385

[2] Boom R, Sol CJ, Salimans MM, Jansen CL, Wertheim-van Dillen PM, van der Noordaa J. 1990. Rapid and simple method for purification of nucleic acids. J Clin Microbiol 28:495–503. doi:10.1128/jcm.28.3.495-503.1990 1691208 PMC269651

[3] Nugent RP, Krohn MA, Hillier SL. 1991. Reliability of diagnosing bacterial vaginosis is improved by a standardized method of gram stain interpretation. J Clin Microbiol 29:297–301. doi:10.1128/jcm.29.2.297-301.1991 1706728 PMC269757

[4] Spackman E, Senne DA, Myers TJ, Bulaga LL, Garber LP, Perdue ML, Lohman K, Daum LT, Suarez DL. 2002. Development of a real-time reverse transcriptase PCR assay for type A influenza virus and the avian H5 and H7 hemagglutinin subtypes. J Clin Microbiol 40:3256–3260. doi:10.1128/JCM.40.9.3256-3260.2002 12202562 PMC130722

[5] Nix WA, Oberste MS, Pallansch MA. 2006. Sensitive, seminested PCR amplification of VP1 sequences for direct identification of all enterovirus serotypes from original clinical specimens. J Clin Microbiol 44:2698–2704. doi:10.1128/JCM.00542-06 16891480 PMC1594621

[6] Lanciotti RS, Kerst AJ, Nasci RS, Godsey MS, Mitchell CJ, Savage HM, Komar N, Panella NA, Allen BC, Volpe KE, Davis BS, Roehrig JT. 2000. Rapid detection of West Nile virus from human clinical specimens, field-collected mosquitoes, and avian samples by a TaqMan reverse transcriptase-PCR assay. J Clin Microbiol 38:4066–4071. doi:10.1128/JCM.38.11.4066-4071.2000 11060069 PMC87542

[7] Lanciotti RS, Calisher CH, Gubler DJ, Chang GJ, Vorndam AV. 1992. Rapid detection and typing of dengue viruses from clinical samples by using reverse transcriptase-polymerase chain reaction. J Clin Microbiol 30:545–551. doi:10.1128/jcm.30.3.545-551.1992 1372617 PMC265106

[8] Helb D, Jones M, Story E, Boehme C, Wallace E, Ho K, Kop J, Owens MR, Rodgers R, Banada P, Safi H, Blakemore R, Lan NTN, Jones-López EC, Levi M, Burday M, Ayakaka I, Mugerwa RD, McMillan B, Winn-Deen E, Christel L, Dailey P, Perkins MD, Persing DH, Alland D. 2010. Rapid detection of Mycobacterium tuberculosis and rifampin resistance by use of on-demand, near-patient technology. J Clin Microbiol 48:229–237. doi:10.1128/JCM.01463-09 19864480 PMC2812290

[9] Brakstad OG, Aasbakk K, Maeland JA. 1992. Detection of Staphylococcus aureus by polymerase chain reaction amplification of the nuc gene. J Clin Microbiol 30:1654–1660. doi:10.1128/jcm.30.7.1654-1660.1992 1629319 PMC265359

[10] Okuma K, Iwakawa K, Turnidge JD, Grubb WB, Bell JM, O’Brien FG, Coombs GW, Pearman JW, Tenover FC, Kapi M, Tiensasitorn C, Ito T, Hiramatsu K. 2002. Dissemination of new methicillin-resistant Staphylococcus aureus clones in the community. J Clin Microbiol 40:4289–4294. doi:10.1128/JCM.40.11.4289-4294.2002 12409412 PMC139674

[11] Chen SM, Dumler JS, Bakken JS, Walker DH. 1994. Identification of a granulocytotropic Ehrlichia species as the etiologic agent of human disease. J Clin Microbiol 32:589–595. doi:10.1128/jcm.32.3.589-595.1994 8195363 PMC263091

[12] Hubert SK, Mohammed JM, Fridkin SK, Gaynes RP, McGowan JE, Tenover FC. 1999. Glycopeptide-intermediate Staphylococcus aureus: evaluation of a novel screening method and results of a survey of selected U.S. hospitals. J Clin Microbiol 37:3590–3593. doi:10.1128/JCM.37.11.3590-3593.1999 10523558 PMC85700

[13] Ginocchio CC, St George K. 2009. Likelihood that an unsubtypeable influenza A virus result obtained with the Luminex xTAG respiratory virus panel is indicative of infection with novel A/H1N1 (swine-like) influenza virus. J Clin Microbiol 47:2347–2348. doi:10.1128/JCM.01027-09 19494066 PMC2708517

[14] Lau SKP, Chan K-H, Yip CCY, Ng T-K, Tsang OTY, Woo PCY, Yuen K-Y. 2009. Confirmation of the first Hong Kong case of human infection by novel swine origin influenza A (H1N1) virus diagnosed using ultrarapid, real-time reverse transcriptase PCR. J Clin Microbiol 47:2344–2346. doi:10.1128/JCM.00924-09 19474268 PMC2708524

[15] Lu X, Whitaker B, Sakthivel SKK, Kamili S, Rose LE, Lowe L, Mohareb E, Elassal EM, Al-sanouri T, Haddadin A, Erdman DD. 2014. Real-time reverse transcription-PCR assay panel for Middle East respiratory syndrome coronavirus. J Clin Microbiol 52:67–75. doi:10.1128/JCM.02533-13 24153118 PMC3911421

[16] Yam WC, Chan KH, Poon LLM, Guan Y, Yuen KY, Seto WH, Peiris JSM. 2003. Evaluation of reverse transcription-PCR assays for rapid diagnosis of severe acute respiratory syndrome associated with a novel coronavirus. J Clin Microbiol 41:4521–4524. doi:10.1128/JCM.41.10.4521-4524.2003 14532176 PMC254368

[17] Carroll KC, Munson E, Butler-Wu SM, Patrick S. 2023. Point-counterpoint: What's in a name? Clinical microbiology laboratories should use nomenclature based on current taxonomy. J Clin Microbiol 61:e0173222. doi:10.1128/jcm.01732-22 36625570 PMC9879091

[18] Dien Bard J, Alby K. 2018. Point-counterpoint: meningitis/encephalitis syndromic testing in the clinical laboratory. J Clin Microbiol 56:e00018-18. doi:10.1128/JCM.00018-18 29343540 PMC5869827

[19] Miller S, Chiu C, Rodino KG, Miller MB. 2020. Point-counterpoint: should we be performing metagenomic next-generation sequencing for infectious disease diagnosis in the clinical laboratory? J Clin Microbiol 58:e01739-19. doi:10.1128/JCM.01739-19 PMC7041574 31619533

[20] Pritt BS, Patel R, Kirn TJ, Thomson RB Jr. 2016. Point-counterpoint: a nucleic acid amplification test for Streptococcus pyogenes should replace antigen detection and culture for detection of bacterial pharyngitis. J Clin Microbiol 54:2413–2419. doi:10.1128/JCM.01472-16 27440817 PMC5035405

[21] Schreckenberger PC, McAdam AJ. 2015. Point-counterpoint: large multiplex PCR panels should be first-line tests for detection of respiratory and intestinal pathogens. J Clin Microbiol 53:3110–3115. doi:10.1128/JCM.00382-15 25762770 PMC4572537

[22] Humphries RM, McAdam AJ. 2025. Reaffirming DEI as a central value of JCM. J Clin Microbiol 63:e0037225. doi:10.1128/jcm.00372-25 40162849 PMC12077182

[23] Ahmed A, Al-Khatib A, Boum Y II, Debat H, Gurmendi Dunkelberg A, Hinchliffe LJ, Jarrad F, Mastroianni A, Mineault P, Pennington CR, Pruszynski JA. 2023. The future of academic publishing. Nat Hum Behav 7:1021–1026. doi:10.1038/s41562-023-01637-2 37443268

[24] Casadevall A, Fang FC. 2024. Fragile science. mBio 15:e0074624. doi:10.1128/mbio.00746-24 38567971 PMC11077952

